# Risk of Fatigue and Anemia in Patients With Prostate Cancer Treated With Novel Oral Anti-androgens: A Meta-Analysis of Randomized Controlled Trials

**DOI:** 10.7759/cureus.21560

**Published:** 2022-01-24

**Authors:** Abdulrahman a Babkoor, Yazeed Aljabri, Ahmad Alzubaidi, Rayan Alhazmi, Zeyad Alsaedi, Faris Alghamdi, Tariq Tamim, Ahmad Aldagani, Irfan Seddiqi, Emad Tashkandi

**Affiliations:** 1 Medicine, Umm Al-Qura University, Makkah, SAU; 2 Pharmacy, King Abdullah Medical City, Makkah, SAU; 3 Community Medicine, Umm Al-Qura University, Makkah, SAU; 4 College of Medicine, Umm Al-Qura University, Makkah, SAU; 5 Oncology Centre, King Abdullah Medical City, Makkah, SAU

**Keywords:** meta-analysis, anemia, fatigue, toxicity, cancer

## Abstract

Novel oral anti-androgens (NOAAs) represent a new class of drugs that are being approved for prostate cancer. However, fatigue and anemia are among the most common treatment-related symptoms. Hence, we conducted a meta-analysis of randomized controlled trials (RCTs) to investigate the relative risks (RRs) of fatigue and anemia associated with NOAAs. PubMed, Cochrane, EMBASE, and abstracts presented at the annual meeting of the American Society of Clinical Oncology and European Society of Clinical Oncology were searched for phase III and V RCTs of NOAAs from January 2000 to March 2020. Safety profile from each selected study was evaluated for all-grade and high-grade fatigue and anemia adverse events. The RRs with 95% confidence intervals (95% CIs) were calculated using random-effects for all-grade and high-grade events.

Our analysis involved 15 RCTs, including 16,795 patients. Overall, 9,177 patients were treated with NOAAs in the experimental arm, whereas 7,095 received a standard of care in the control arm. The RR of all-grade and high-grade fatigue was 1.26 (95% CI 1.15-1.38) and 1.24 (95% CI 0.83-1.84), and that of all-grade and high-grade anemia was 0.81 (95% CI 0.77-1.19) and 0.81 (95% CI 0.61-1.06), respectively. Our findings suggest that NOAAs are associated with an increased risk of fatigue but decreased risk of anemia. Patients should be frequently monitored to identify adverse events to improve oncological outcomes and optimize the overall treatment efficacy and safety. Not all the RCTs addressed fatigue and anemia simultaneously as side effects of NOAA treatment.

## Introduction and background

Prostate cancer (PC) is the second most common malignancy in men and the fifth leading cause of cancer-related death in men worldwide. According to GLOBOCAN 2020, there are approximately 1.4 million new PC cases and 375,000 PC-associated deaths worldwide [1]. PC is an androgen-dependent malignancy; therefore, androgen deprivation therapy (ADT) is the backbone treatment for newly diagnosed advanced or recurrent PC [2]. Anti-androgens inhibit testosterone production in the testes and prostate tumors by blocking androgen receptors [3-4]. The first generation of these drugs, including bicalutamide, nilutamide, and flutamide [5] that were used as therapy for the management of advanced PC since 1941 [6], did not fully block androgen receptor activity. The second generation showed superior efficacy and potency and thus became the standard of care (SOC) [7]. Currently, the US Food and Drug Administration has permitted four novel oral anti-androgens (NOAAs): abiraterone acetate, enzalutamide, and recently, apalutamide and darolutamide [7-8]. These agents prevent testicular and extragonadal testosterone synthesis either by inhibiting the key enzyme in androgen production, cytochrome P450 17A1, as abiraterone acetate does, or by inhibiting the nuclear translocation of androgen receptors in the prostate, as enzalutamide, darolutamide, and apalutamide do [7].

NOAAs have clearly improved patient survival in several clinical trials and reduced PC-associated deaths. Moreover, several new studies have been conducted, and several are ongoing to test NOAA efficacy and safety for PC in different settings, either alone or in combination, with some promising results. Because these agents are new, it is crucial to understand their adverse event (AE) profile because patients can be using them for many years. Fatigue and anemia are observed as side effects in this generation and can be debilitating, leading to dose reduction or discontinuation of an effective agent. Furthermore, these conditions can be attributed to underlying malignancy or medical disorders, and in some situations, to drug-related complications. In this systematic review and meta-analysis, we aimed to investigate the risk of treatment-related fatigue and anemia in patients with PC.

## Review

Materials and methods

Protocol Registration

The protocol for this systematic review was registered with the International Prospective Register of Systematic Reviews (PROSPERO) (CRD42020197743).

Search Method and Study Election

We searched PubMed, Cochrane Library, and EMBASE from January 1, 2000, to March 1, 2020, for randomized controlled trials (RCTs) that compared NOAAs with standard care treatment such as ADT in men who were diagnosed histologically as having PC regardless of the stage of cancer and irrespective of race. We also searched abstracts and presentations from relevant conference proceedings, including the American Society of Clinical Oncology and the European Society for Medical Oncology, until March 1, 2020. Additionally, we searched the clinical trial registration website (http://www. ClinicalTrials.gov) to collect information on registered prospective trials. Studies that met the following eligibility criteria were included: articles published in English language, prospective phase III RCTs design in PC, studies in which participants were assigned to treatment with NOAAs or control (placebo, chemotherapy, or other therapies), and studies with available safety data reporting AEs. Retrospective studies and phase I and phase II trials were excluded due to the lack of control groups. In addition, case reports, editorials, letters, review articles, and conference abstracts were excluded.

Keywords for the literature search included published randomized trials, prostate cancer, prostate adenocarcinoma, small cell prostate cancer, prostate neoplasm, apalutamide, darolutamide, abiraterone, enzalutamide, antiandrogen, testosterone blockers, androgen antagonists, and androgen deprivation therapy. All RCTs that compared NOAAs with SOC in patients with PC were selected. For studies with more than one publication, data on all outcomes were extracted from the most recent and updated versions and included in the analysis. The primary outcome was the incidence of all-grade and high-grade fatigue 3 or 4, which was defined according to the Common Terminology Criteria for Adverse Events (CTCAE) of the National Cancer Institute (NCI-CTC). The NCI CTCAE is an expressive terminology that can be applied for AE reporting [9]. The secondary outcome was the incidence of all-grade and high-grade anemia 3 or 4 [10]. 

Data Extraction and Quality Assessment

This study observed the Ideal Reporting Items for Systematic Reviews and Meta-analyses (PRISMA) statement [11]. A protocol was created in advance and uploaded to the PROSPERO online platform. Two reviewers (AB and ZS) made data extraction independently, with disagreements resolved by a third reviewer (ET).

The study name, first author, year of publication, journal, trial number or abbreviation, study design, median age, number of patients, disease stage non-metastatic castrate-resistant PC (nmCRPC) vs. metastatic hormone-sensitive PC (mHSPC) or metastatic castrate-resistant PC (mCRPC), Eastern Cooperative Oncology Group (ECOG) Performance Status that determines the ability of patients to endure therapies in serious illness, precisely with chemotherapy (0 vs. 1-2), intervention and comparison group, dosage, duration of therapy, follow-up, incidence of fatigue and proportion of high-grade AEs (grade 3 or 4), incidence of anemia and proportion of high-grade AEs (grade 3 or 4), and RRs with 95% CIs were extracted. Data on the incidence of all-grade and high-grade (3 or 4) fatigue and anemia were collected from each study’s safety profile or supplemental material.

We assessed the methodological quality of each study for risk of bias using the criteria recommended in the Cochrane Handbook across the following domains: sequence generation, allocation concealment, blinding of participants, blinding of personnel and outcome assessors, incomplete outcome data, selective outcome reporting, and other potential threats to validity. Each aspect was evaluated, with an assessment index associated with the risk of bias classified as low or high risk of bias or as unclear risk of bias according to the criteria in the Cochrane Handbook [12]. Disagreements in the risk of bias assessment between the two reviewers were settled through discussion, or if needed, by a third reviewer. We presented the results of the risk of bias assessment in a “risk of bias.”

Statistical analysis

The numbers of patients for each AE and those treated with each NOAA were calculated to determine the incidence. In addition, the proportions of patients who experienced adverse outcomes were recorded. The relative risks (RRs) and 95% confidence intervals (95% CIs) of each AE were calculated in patients assigned to NOAAs vs. SOC. A sensitivity analysis was performed by sequential omission of individual studies to assess the stability of the results. The RRs were calculated using a random-effects model weighted by the number of treated patients [13]. Heterogeneity was evaluated using Cochrane’s Q statistic, and inconsistency was quantified with the I2 statistic (100%×[Q-df]/Q), which represents the percentage of total variation across studies. An I2 value of >50% was considered indicative of substantial heterogeneity [12]. Pre-specified subgroup analyses were also conducted for different NOAAs used and tumor types (non-metastatic vs. hormone-sensitive vs. metastatic castrate-resistant PC). Publication bias was assessed using a funnel plot [14]. A two-tailed P-value of <0.05 was considered statistically significant. All statistical analyses were performed using Review Manager 5.3, 2014.

Results

Search Results

**Figure 1:**
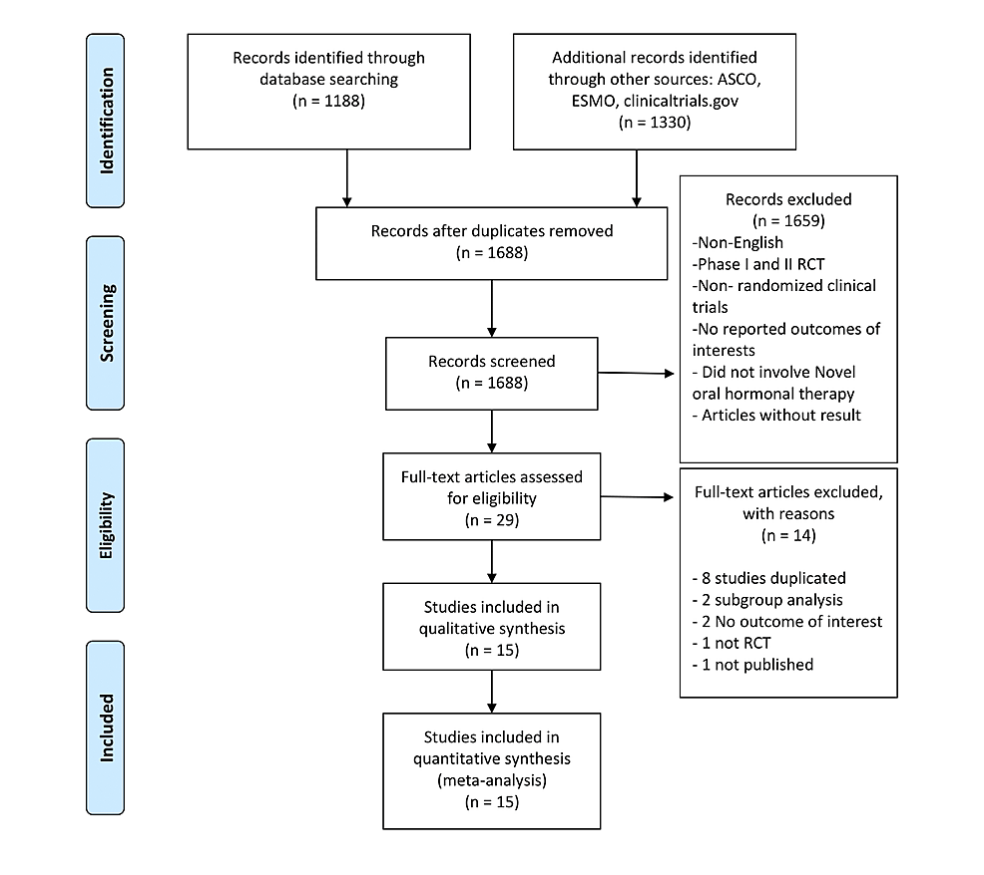
Flow diagram illustrating the selection of included studies. ASCO: American Society of Clinical Oncology; ESMO: European Society for Medical Oncology; RCT: randomized controlled trial

The search and review of the reference lists identified a total of 2,518 studies for screening (Figure 1). After removal of duplicates that were excluded automatically by Mendeley, 1,688 articles were identified. After screening and eligibility assessment, a total of 1,659 irrelevant papers (case reports, editorials, letters, review articles, conference abstracts, and phase I or phase II trials) were excluded. Finally, among the 29 remaining publications, eight were manually excluded because they were duplicates, two were subgroup analyses, two had no outcome of interest, one was not an RCT, and one was unpublished. Finally, 15 RCTs (n = 16,795 patients) were considered eligible for meta-analysis (Table 1).

**Table 1:** Characteristics of included trials. n: number; SOC: standard of care; mHSPC: metastatic hormone-sensitive prostate cancer; mCRPC: metastatic castrate-resistant prostate cancer; nmCRPC: non-metastatic castrate-resistant prostate cancer; ADT: androgen deprivation therapy; CTCAE: common terminology criteria for adverse events; NA: not available

Author/year	Phase	Median age, years	Subjects, n	Clinical condition	Treatment arm	Control arm	Median follow-up duration, months	CTCAE version
Armstrong et al., 2019- ARCHES [15]	3	70	1150	mHSPC	Enzalutamide 160 mg + ADT	Placebo + ADT	14.4	4
Chi et al., 2019-TITAN [16]	3	69	1052	mHSPC	Apalutamide 240 mg + ADT	Placebo + ADT	22.7	4
Fizazi et al., 2019-LATITUDE [17]	3	68	1199	mHSPC	Abiraterone 1000 mg + Prednisone 5 mg + ADT	Placebo + ADT	51.8	4
Fizazi et al, 2019- ARAMIS [18]	3	74	1509	nmCRPC	Daralutamide 300 mg	Placebo + ADT	17.9	4
Davis et al., 2019 - ENZAMET [19]	3	69	1125	mHSPC	Enzalutamide 160 mg	Bicalutamide, nilutamide, or flutamide	34	4
Smith et al., 2018- SPARTAN [20]	3	74	1207	nmCRPC	Apalutamide 240 mg	Placebo	20.3	4
Hussain et al., 2018 - PROSPER [21]	3	74	1401	nmCRPC	Enzalutamide 160 mg	Placebo	18.5	4
Beer et al., 2017- PREVAIL [22]	3	72	1717	mCRPC	Enzalutamide 160 mg	Placebo	22	4
de Bono et al., 2011 - COU-AA-301 [23]	3	69	1195	mCRPC	Abiraterone 1000 mg + prednisone 5 mg	Placebo + prednisone 5 mg	12.8	3
Scher et al., 2012 AFFIRM [24]	3	69	1199	mCRPC	Enzalutamide 160 mg	Placebo	14.4	4
Sun et al., 2016 [25]	3	68	214	mCRPC	Abiraterone 1000 mg + prednisone 5 mg	Placebo + prednisone 5 mg	12.9	NA
Ye et al., 2017 [26]	3	71	313	mCRPC	Abiraterone 1000 mg + prednisone 5 mg	Placebo + prednisone 5 mg	3.9	NA
Ryan et al., 2013 COU-AA-302 [27]	3	70	1088	mCRPC	Abiraterone 1000 mg + prednisone 5 mg	Placebo + prednisone 5 mg	22.2	NA
James et al., 2017 - STAMPEDE [28]	2-3	67	1917	mHSPC	Abiraterone 1000 mg + prednisone 5 mg + ADT	ADT		4
Attard et al., 2018- PLATO [29]	4	72	509	mCRPC	Enzalutamide 160 mg + abiraterone 1000 mg + prednisone 5 mg	Placebo + abiraterone 1000 mg + prednisone 5 mg	5.7	4

Study quality

**Figure 2:**
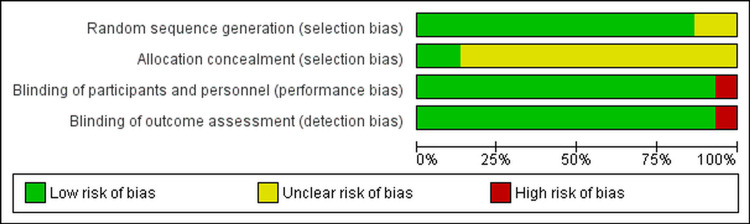
Risk of bias graph.

**Figure 3:**
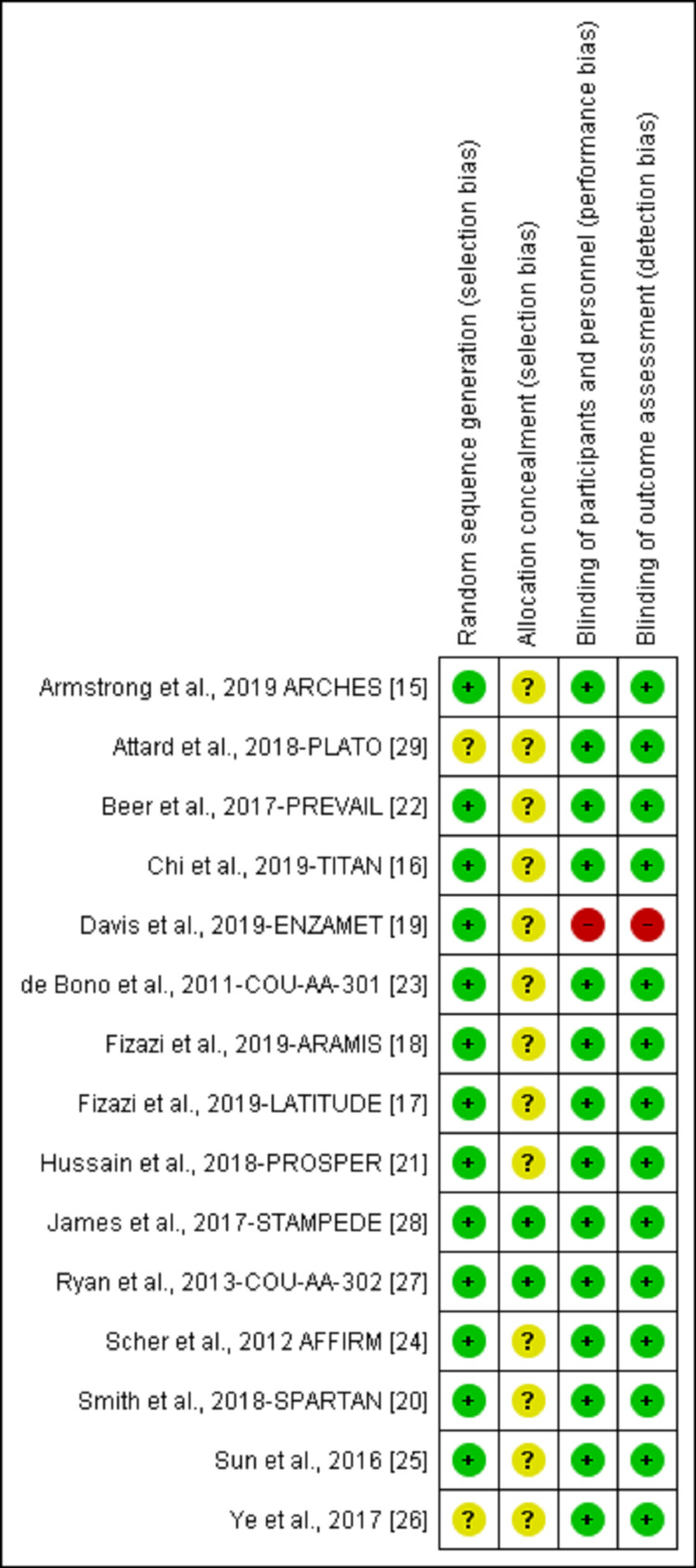
Risk of bias summary. [15-29]

The included studies were published as full manuscripts between 2011 and 2019. All included trials were randomized in multicenter, with 13 phase III trials (Armstrong et al., 2019 [15], Chi et al., 2019 [16], Fizazi et al., 2019 LATITUDE [17], Fizazi et al., 2019 ARAMIS [18], Davis et al., 2019 [19], Smith et al., 2018 [20], Hussain al., 2018 [21], Beer et al., 2017 [22], De Bono et al., 2011 [23], Scher et al., 2012 [24], Sun et al., 2016 [25], Ye et al., 2017 [26], Ryan et al., 2013) [27], one phase II/III trial (James et al., 2017) [28], and one phase IV trial (Attard et al., 2018) [29]. Using the Cochrane collaboration tool for risk of bias classification, a tool to assess the risk of bias in randomized trials, we found that the quality of the included studies was generally good and fair, with all trials categorized as being of good quality (Figures 2, 3). All 12 trials reported AEs according to the National Cancer Institute’s CTCAE version 3 or 4 criteria.

Patients

A total of 16,795 patients were included in the meta-analysis; among them, 9,177 patients were treated with NOAAs in the experimental arms, while 7,095 received SOC in the control arms. The population included were patients with nmCRPC in three studies (Smith et al., 2018 [20], Hussain et al., 2018 [21], and Fizazi et al., 2019 ARMIS [18]) and those with mHSPC in five studies (Chi et al., 2019 [16], Fizazi et al., 2019 LATITUDE [17], Davies et al. (2019) [19], James et al. (2017) [28], and Armstrong et al., 2019 [15]). The remaining seven studies included patients with mCRPC.

NOAAs were compared against a placebo alone in four studies (Beer et al., 2017 [22], Hussain et al., 2018 [21], Scher et al., 2012 [24], Smith et al., 2018 [20]), a placebo plus ADT in one study (Fizazi et al., 2019) [17-18], a placebo plus ADT in four studies (Chi et al., 2019 [16], Fizazi et al., 2019 LATITUDE [17], Fizazi et al., 2019 ARMIS [18]), a placebo and prednisone in four studies (de Bono et al., 2011 [23], Ryan et al., 2013 [27], Sun et al., 2016 [25], Ye et al., 2017 []26), a non-steroidal oral antiandrogen in one study (Davies et al., 2019) [19], and an ADT alone in one study (James et al., 2017) [28].

The NOAA drugs and doses were apalutamide (240 mg) in two studies (Chi et al., 2019 [16], Smith et al., 2018 [20]); abiraterone (1000 mg) was combined with prednisone 5 mg in five studies (de Bono et al., 2011 [23], Fizazi et al., 2019 [17-18], James et al., 2017 [28], Ryan et al., 2013 [27], Sun et al., 2016 [25], Ye et al., 2017 [26]); daralutamide (300 mg) was used in one study (Fizazi et al., 2019 [17-18]); and enzalutamide (160 mg) was used in six studies (Armstrong et al., 2019 [15], Attard et al., 2018 [29], Beer et al., 2017 [22], Davies et al., 2019 [19], Hussain et al., 2018 [21], Scher et al., 2012 [24]). All included studies reported safety data on fatigue and anemia, which were included in the analysis.

Incidence and relative risk of fatigue

**Figure 4:**
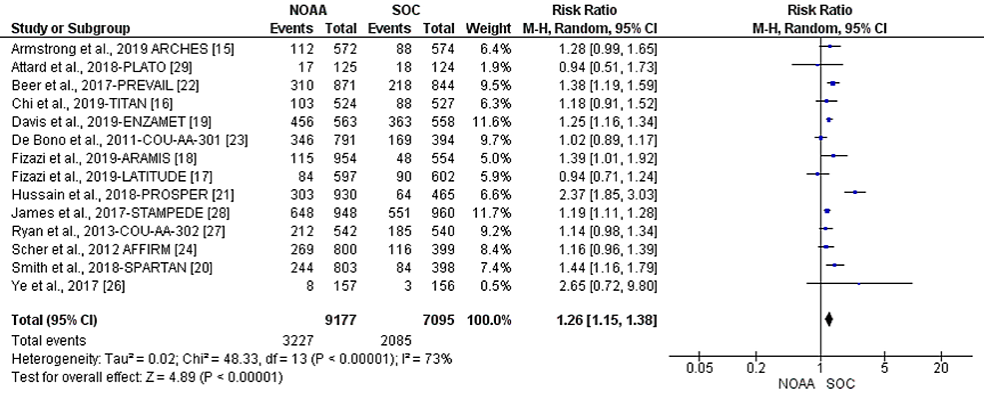
Relative risk for all-grade fatigue. NOAA: novel oral anti-androgen; SOC: standard of care [15-24,26-29]

**Figure 5:**
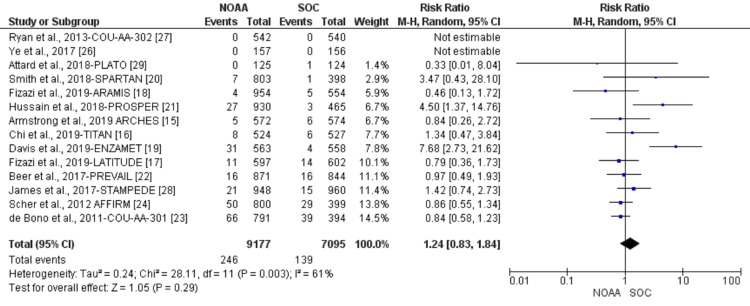
Relative risk for high-grade fatigue. NOAA: novel oral anti-androgen; SOC: standard of care [15-24,26-29]

Using a random-effect model, the RR of all-grade fatigue revealed a 26% higher risk of fatigue in NOAAs than in SOC (RR 1.26, 95% CI 1.15-1.38, heterogeneity: I2= 73%, P<0.00001, Figure 4). The RR for high-grade fatigue (grade 3-5) revealed a 24% higher risk of fatigue in NOAAs than in SOC (RR 1.24, 95% CI 0.83-1.84, heterogeneity: I2= 61%, P=0.003, Figure 5).

Incidence and relative risk of anemia

**Figure 6:**
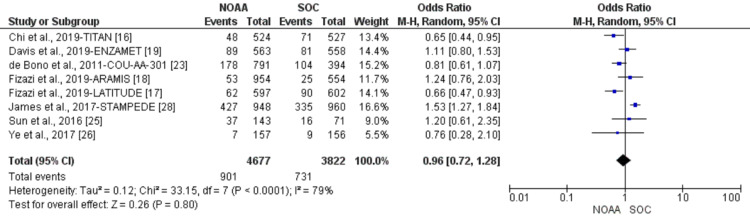
Relative risk for all-grade anemia. NOAA: novel oral anti-androgen; SOC: standard of care [16-19,23,25-26,28]

**Figure 7:**
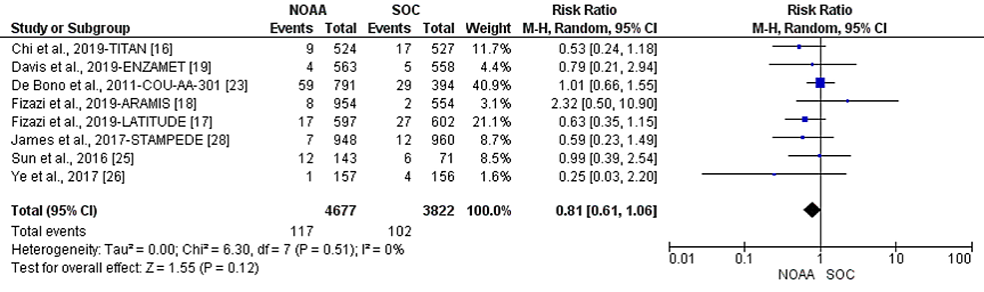
Relative risk for high-grade anemia in patients treated with novel oral anti-androgens (NOAAs) or standard of care (SOC). NOAA: novel oral anti-androgen; SOC: standard of care [16-19,23,25-26,28]

Using a random-effect model, the RR of all-grade anemia revealed 4% less risk of anemia in NOAAs than in SOC (RR 0.96, 95% CI 0.77-1.19, heterogeneity: I2= 77%, P<0.0001, Figure 6). The RR for high-grade anemia (grade 3-5) revealed 19% less risk of anemia in NOAAs than in SOC (RR 0.81, 95% CI 0.61-1.06, heterogeneity: I2= 0%, P=0.51, Figure 7).

Subgroup analysis

**Figure 8:**
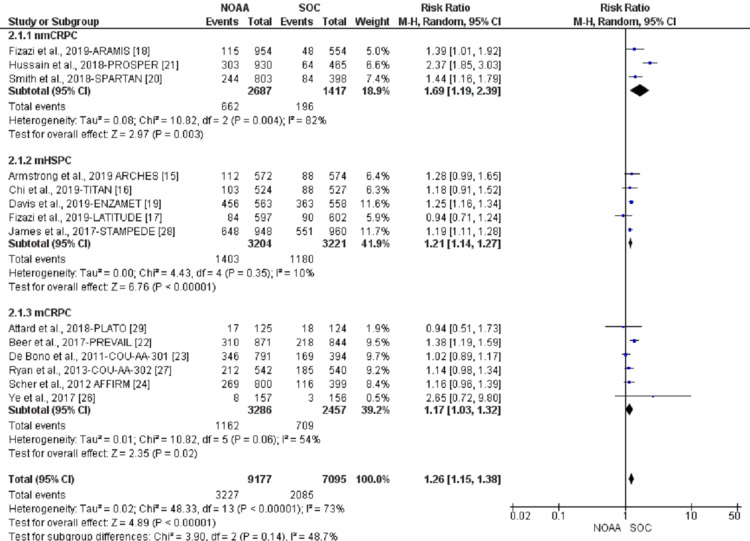
All-grade fatigue subgroup analysis of non-metastatic castrate-resistant prostate cancer (nmCRPC) vs. metastatic hormone-sensitive prostate cancer (mHSPC) or metastatic castrate-resistant prostate cancer (mCRPC) NOAA: novel oral anti-androgen; SOC: standard of care; nmCRPC: non-metastatic castrate-resistant prostate cancer; mHSPC: metastatic hormone-sensitive prostate cancer; mCRPC: metastatic castrate-resistant prostate cancer [15-24,26-29]

Pre-specified subgroup analyses were conducted according to different stages of PC: nmCRPC vs. mHSPC or mCRPC. nmCRPC had the highest risk of all-grade fatigue (69%; RR 1.69, 95% CI 1.19-2.39, I2= 82%), followed by mHSPC (21%; RR 1.21, 95% CI 1.14-1.27, I2= 10%) and mCRPC (17%; RR 1.17, 95% CI 1.03-1.32, I2= 54%, Figure 8).

**Figure 9:**
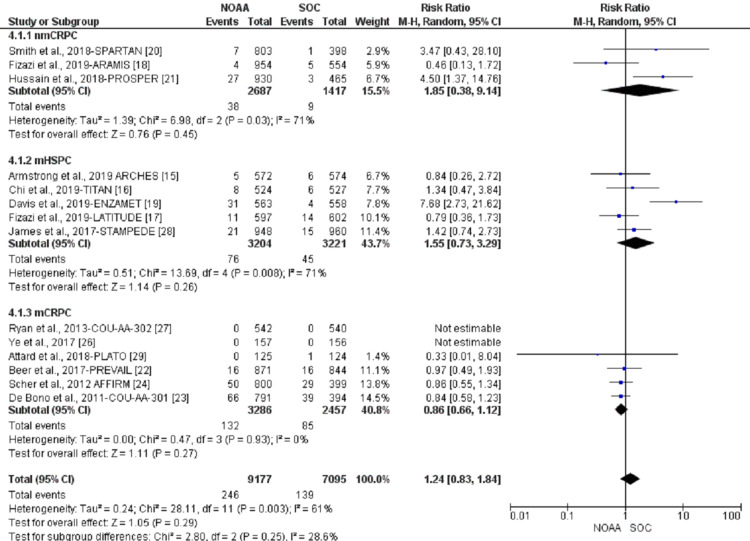
High-grade fatigue subgroup analysis of non-metastatic castrate-resistant prostate cancer (nmCRPC) vs. metastatic hormone-sensitive prostate cancer (mHSPC) or metastatic castrate-resistant prostate cancer (mCRPC). NOAA: novel oral anti-androgen; SOC: standard of care; nmCRPC: non-metastatic castrate-resistant prostate cancer; mHSPC: metastatic hormone-sensitive prostate cancer; mCRPC: metastatic castrate-resistant prostate cancer [15-24,26-29]

nmCRPC had the highest risk of high-grade fatigue (85%; RR 1.85, 95% CI 0.38-9.14, I2= 71%), followed by mHSPC (55%; RR 1.55, 95% CI 0.73-3.29, I2= 71%), and mCRPC (14%; RR 0.86, 95% CI 0.66-1.12, I2= 0%, Figure 9).

**Figure 10:**
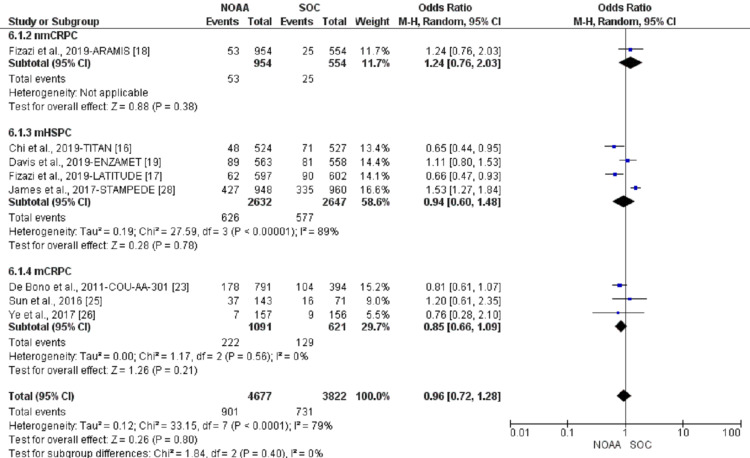
All-grade anemia subgroup analysis of non-metastatic castrate-resistant prostate cancer (nmCRPC) vs. metastatic hormone-sensitive prostate cancer (mHSPC) or metastatic castrate-resistant prostate cancer (mCRPC). NOAA: novel oral anti-androgen; SOC: standard of care; nmCRPC: non-metastatic castrate-resistant prostate cancer; mHSPC: metastatic hormone-sensitive prostate cancer; mCRPC: metastatic castrate-resistant prostate cancer [15-18,22,24-25,27]

nmCRPC had the highest risk of all-grade anemia (23%; RR 1.23, 95% CI 0.77-1.96, I2= 0%), followed by mHSPC (8%; RR 0.92, 95% CI 0.65-1.30, I2= 88%), and mCRPC (12%; RR 0.88, 95% CI 0.73-1.07, I2= 0%, Figure 10).

**Figure 11:**
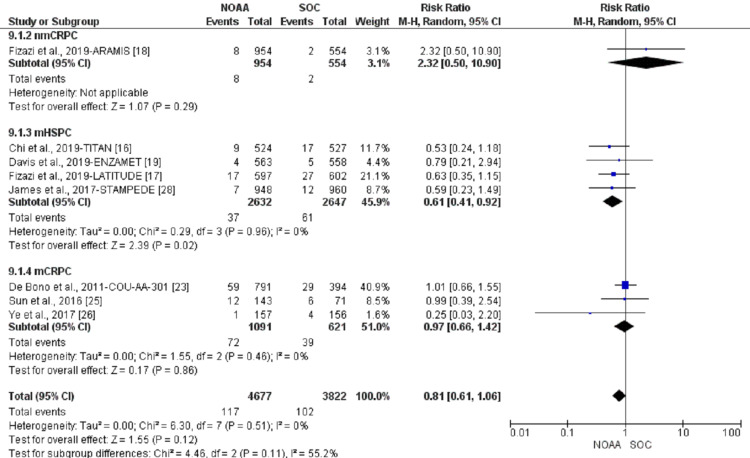
High-grade anemia subgroup analysis of non-metastatic castrate-resistant prostate cancer (nmCRPC) vs. metastatic hormone-sensitive prostate cancer (mHSPC) or metastatic castrate-resistant prostate cancer (mCRPC). NOAA: novel oral anti-androgen; SOC: standard of care; nmCRPC: non-metastatic castrate-resistant prostate cancer; mHSPC: metastatic hormone-sensitive prostate cancer; mCRPC: metastatic castrate-resistant prostate cancer [16-19,23,25-26,28]

nmCRPC was suggested to have the highest risk of high-grade anemia (132%; RR 2.32, 95% CI 0.50-10.90, I2= 0%), followed by mHSPC (39%; RR 0.61, 95% CI 0.41-0.92, I2= 0%), and mCRPC (3%; RR 0.97, 95% CI 0.66-1.42, I2= 0%, Figure 11).

Sensitivity analysis

**Figure 12:**
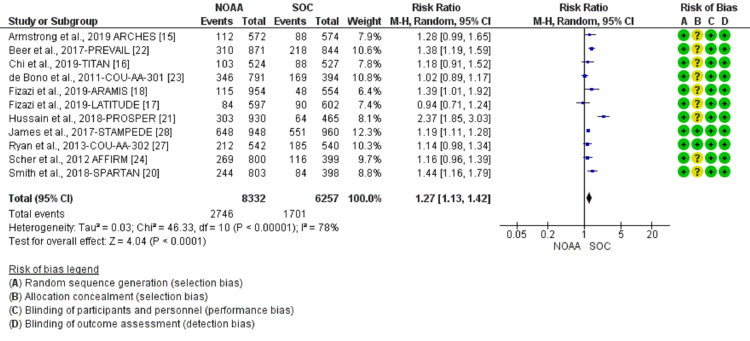
Sensitivity analysis for all-grade fatigue. NOAA: novel oral anti-androgen; SOC: standard of care [15-18,20-23,28]

**Figure 13:**
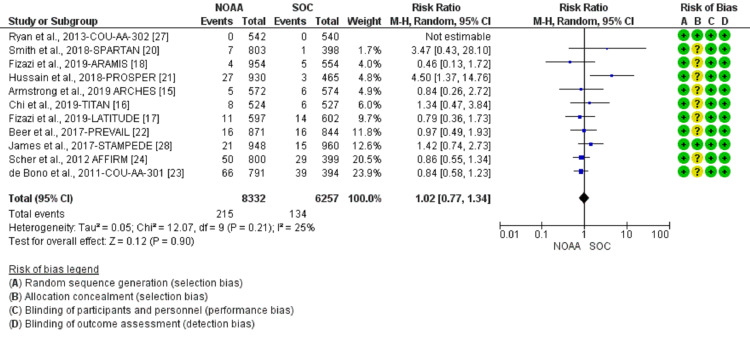
Sensitivity analysis for high-grade fatigue. NOAA: novel oral anti-androgen; SOC: standard of care [15-18,20-23,27-28]

**Figure 14:**
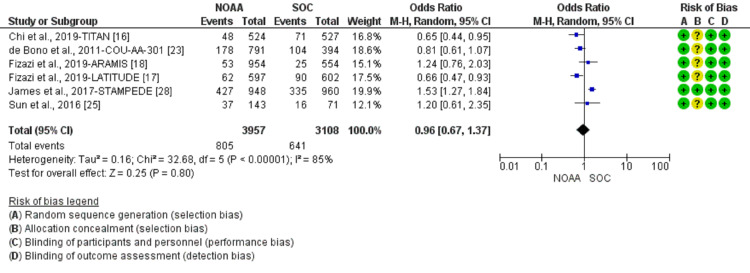
Sensitivity analysis for all-grade anemia. NOAA: novel oral anti-androgen; SOC: standard of care [16-18,20-23,25,28]

**Figure 15:**
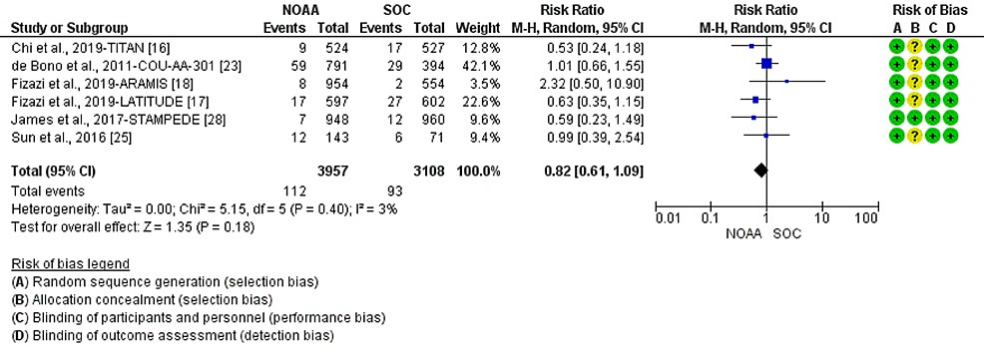
Sensitivity analysis for high-grade anemia. NOAA: novel oral anti-androgen; SOC: standard of care [16-18,20-23,25,28]

The effect of the risk of bias on the results was examined using sensitivity analysis, limiting the analysis to trials at low risk of bias for random sequence generation and blinding. Studies with a high risk of bias or uncertain risk were removed (blinding in Davis et al., 2019 [19], and random sequence generation in Attard et al., 2019 [29], Ye et al., 2017 [26]). All-grade fatigue showed no significant change (Figures 12-15).

Publication bias

**Figure 16:**
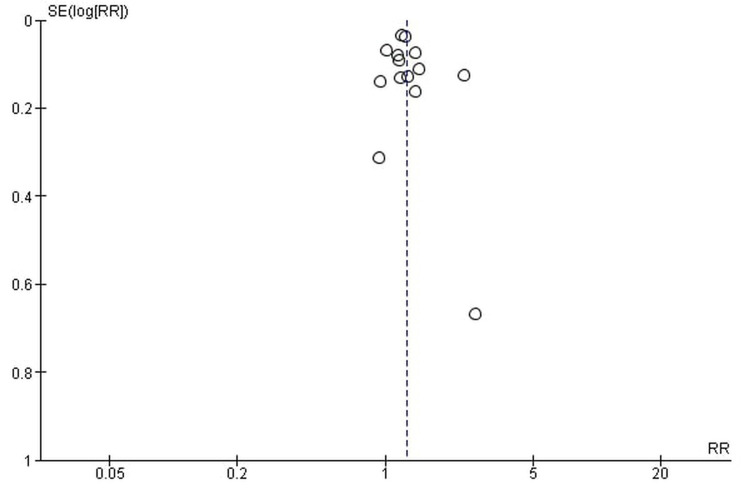
All-grade fatigue.

**Figure 17:**
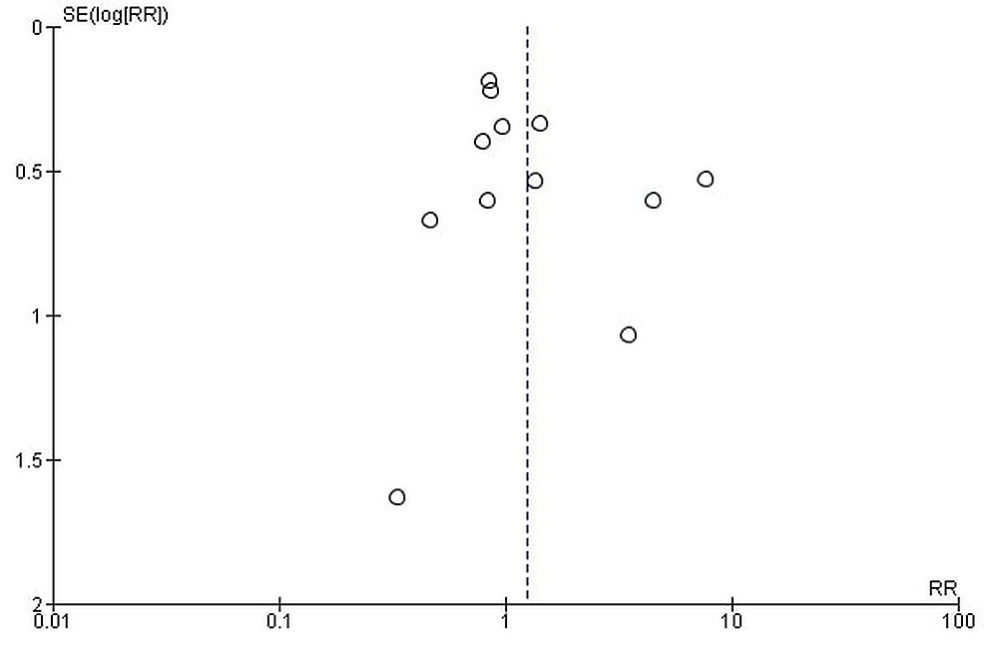
High-grade fatigue.

**Figure 18:**
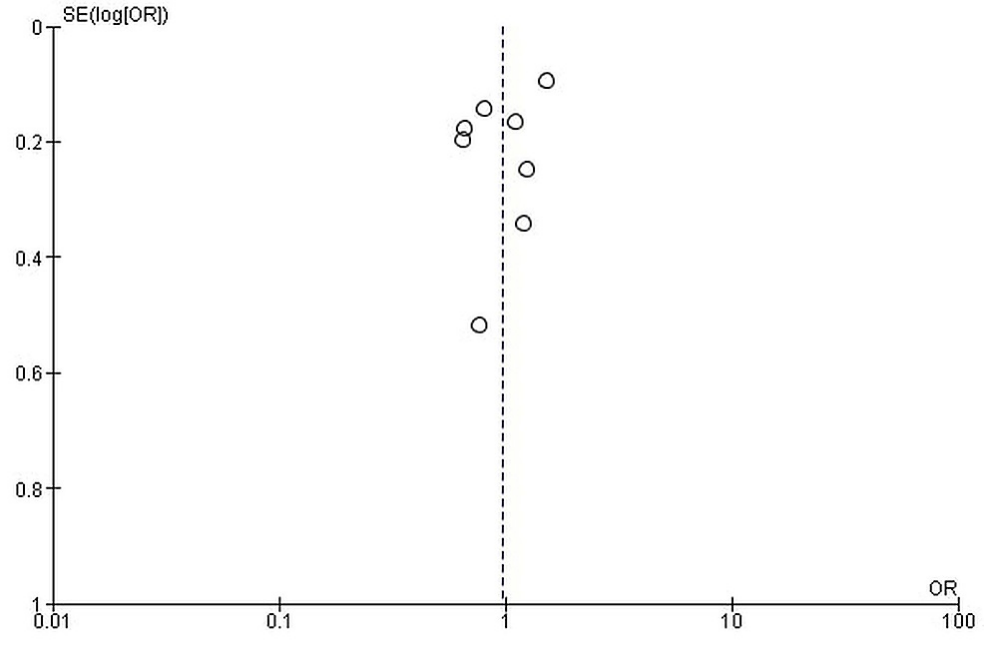
All-grade anemia.

**Figure 19:**
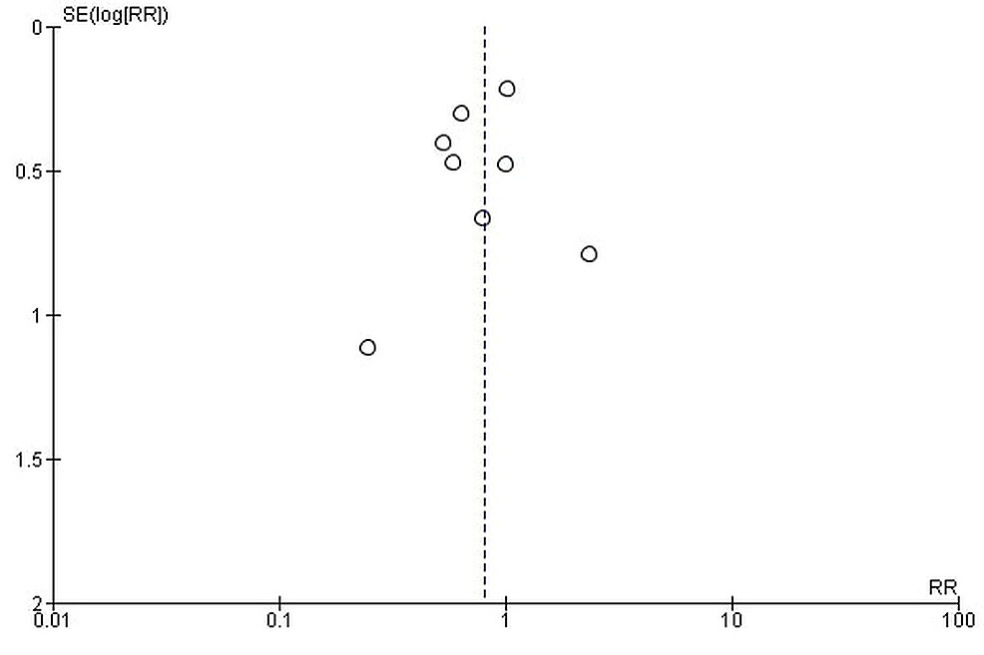
High-grade anemia.

The funnel plots showed symmetrical shapes, which suggested no publication bias (Figures 16-19).

 

Discussion

To the best of our knowledge, this is the first meta-analysis to investigate the incidence of fatigue and anemia associated with the use of NOAAs in PC. The RR of all-grade and high-grade fatigue associated with NOAAs was increased by 26% and 24%, respectively, and the RR of all-grade and high-grade anemia was decreased by 4% and 19%, respectively. Moreover, the RR of fatigue remained significant after excluding trials that had active NOAAs in the control arms and different stages such as nmCRCP, mCRPC, and mHSPC. Fatigue is the most distressing symptom of advanced cancer, affecting patients’ quality of life and limiting their activities; treatment modalities such as chemotherapy or radiotherapy increase its occurrence [30]. In this review, the use of NOAAs significantly increased the risk of both all- and high-grade fatigue. Enzalutamide therapy has been reported to be associated with a significantly increased risk of all- and high-grade fatigue by 29% and 50%, respectively [31]. A meta-analysis found that the risk of high-grade fatigue was higher by 25% in patients treated with new hormonal therapies; however, the difference was not significant [32]. A network meta-analysis found that patients who received NSAAs (non-steroidal antiandrogen) had an increased likelihood of experiencing high-grade fatigue by 21%, and fatigue appeared to be more pronounced in patients receiving enzalutamide [33]. These results are consistent with the findings of our meta-analysis in that NOAAs increased the risk of all- and high-grade fatigue in PC. Anticancer therapy in advanced PC causes anemia and reduces the quality of life [33]. In our review, the use of NOAAs significantly decreased the risk for all-grade anemia and insignificantly decreased the risk of high-grade anemia compared to SOC. To the best of our knowledge, this is the first meta-analysis to measure the effect of NOAAs (enzalutamide, darolutamide, apalutamide, and abiraterone) on the incidence of anemia.

The strengths of this meta-analysis were the 15 studies that included a large number of participants. In addition, four NOAA treatments were included, whereas other studies compared one or two NOAA treatments; therefore, this is the first study to compile all NOAAs. Among the limitations is that not all the RCTs addressed both fatigue and anemia simultaneously as side effects of NOAA treatment, wherein the majority of studies addressed fatigue only. In addition, despite significant results, there was relatively high heterogeneity in all-grade and high-grade fatigue and anemia.

## Conclusions

In conclusion, our study shows that NOAAs are associated with a significant increase in the risk of all-grade and high-grade fatigue AEs. The use of NOAAs significantly decreased the risk for all-grade anemia and insignificantly decreased the risk of high-grade anemia compared to SOC. Fatigue is considered the most common distressing symptom among patients with advanced cancer. There are very few options currently available to manage cancer-related fatigue (CRF), non-pharmacological as lifestyle modifications or pharmacological which aim to reduce side effects of treatment as stimulants, such as methylphenidate. This can be debilitating for some patients; therefore, practicing oncologists need to monitor this AE regularly to achieve the best oncological outcomes, Future studies need to address the fatigue issue in patients to increase awareness regarding this AE when using NOAAs, to avoid non-compliance with medications.
